# Supplementary material for: Cryo-EM structures of full-length integrin αIIbβ3 in native lipids
Source: Nat Commun. 2023 Jul 13;14:4168. doi: 10.1038/s41467-023-39763-0 (PMC10345127; doi:10.1038/s41467-023-39763-0)
Supplement: Supplementary file 1 — Supplementary Information [file 41467_2023_39763_MOESM1_ESM.pdf]

Supplementary Information

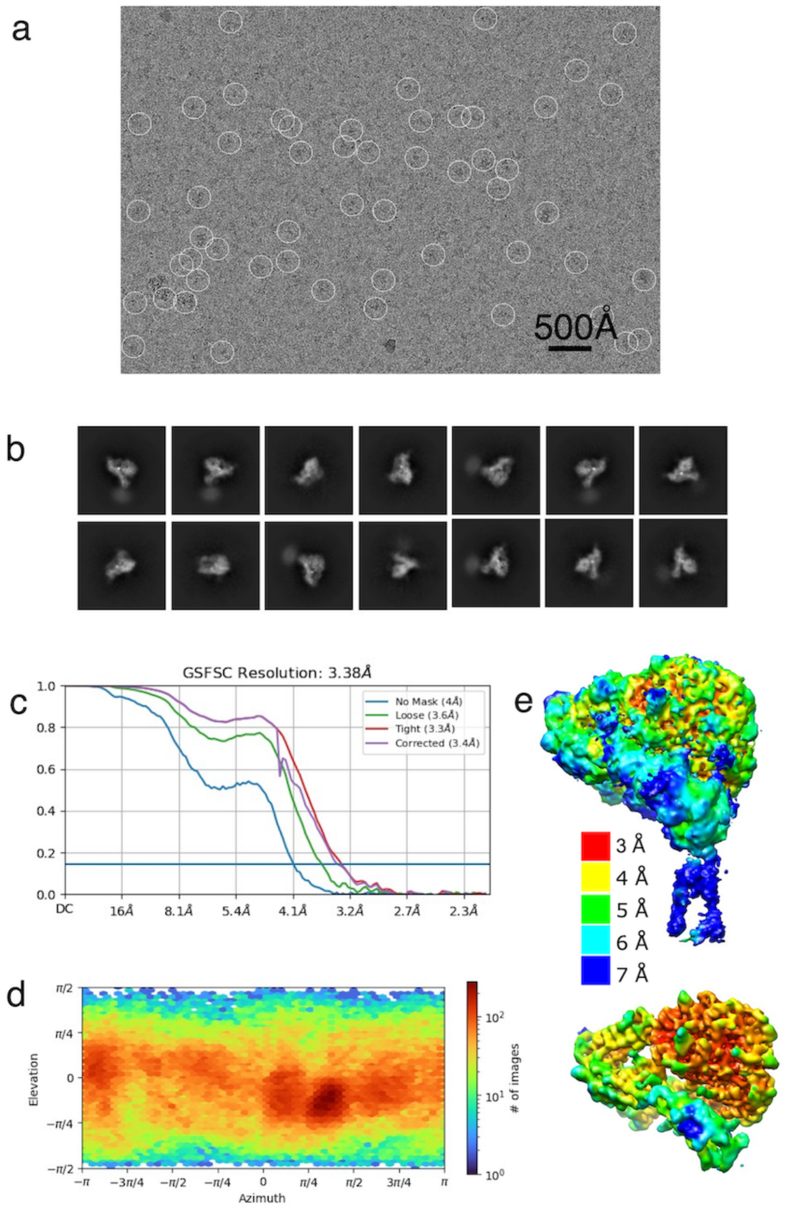

Supplementary Fig. 1. EM processing of full-length αIIbβ3. a) A representative cryo-EM micrograph with selected particles used for the reconstruction indicated with white circles. This micrograph is one of 11,929 micrographs with selected particles. The scale bar at the lower right shows 500 Å. b) Reference-free class averages generated from selected particles. The averages clearly show diffuse density for the transmembrane region. c) Fourier-shell correlation curves of the two half maps for the final round of refinement. The 0.143 cutoff criterion has been used to determine the resolution. d) Heat map showing the orientation distribution of particles. Euler angles are indicated along the X and Y coordinates. Color encoding for particle numbers is shown on the right. e) Color-coded local resolution maps. The top and bottom maps show different isosurface values**.** Colors encode graded resolutions between 3.0 Å (red) and 7.0 Å (blue).

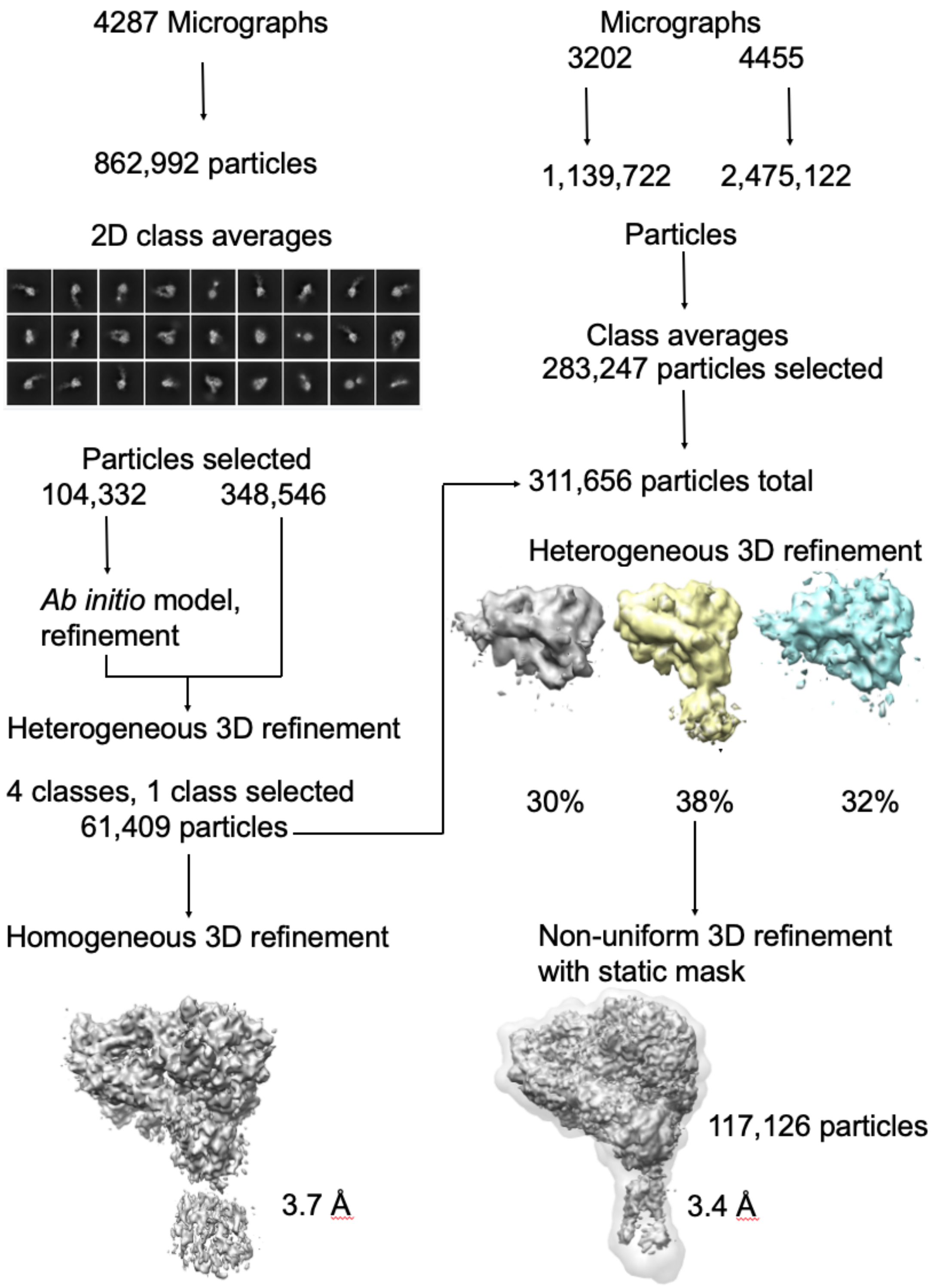

Supplementary Fig. 2. Flowchart for EM analysis of full-length inactive αIIbβ3 with Cryosparc. Flowchart for processing of the native integrin. Details are provided in the methods section. The final map at 3.4 Å is displayed with the static mask used for refinement.

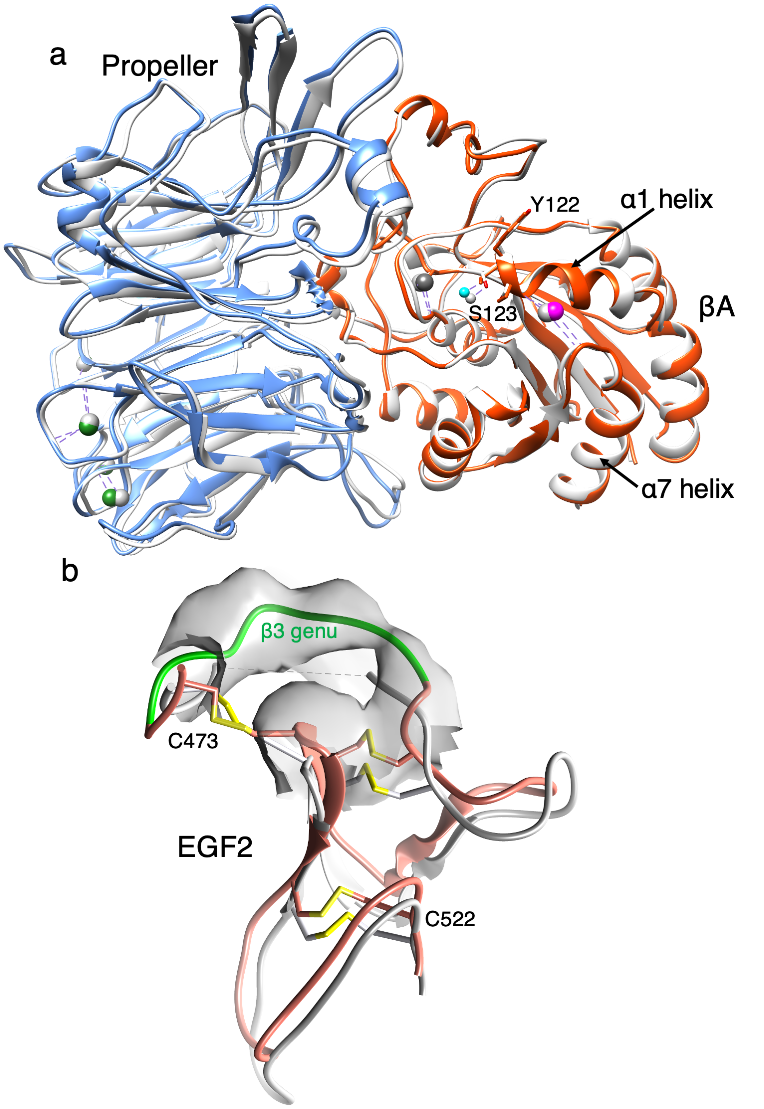

Supplementary Fig. 3. **Structures of the head domain and the β genu of full-length inactive αIIbβ3.** (a) Ribbon diagrams showing the structure of the head (propeller in light blue and βA in orange-red) from full-length αIIbβ3 superimposed onto that from the crystal structure of the recombinant ectodomain (3fcs.pdb). The αIIbβ3 head of the full-length integrin assumes the inactive conformation, reflected by the ADMIDAS Ca^{2+}-mediated stabilizing link between the activation-sensitive α1 and α7 helices of the βA domain, preventing the inward movement of the α1 helix (reported by Y122) and the direct coordination of the MIDAS metal by S123 of the α1 helix. Metal ions at ADMIDAS, MIDAS, and LIMBS are shown as magenta, cyan, and gray spheres, respectively, for the cryo-EM structure and gray spheres for the crystal structure. The four Ca^{2+} ions at the bottom of the propeller are shown as dark green and gray spheres in the respective structures. (b) Ribbon diagrams of the EGF2 domain from full-length αIIbβ3 (in dark red with the β3 genu in green) superposed onto that from 3fcs.pdb (in gray). The main chain of the β3 genu with the surrounding EM density is missing in 3fcs.pdb. The four disulfide bonds in EGF2 are shown in yellow, with some of the cysteines (C) labeled.

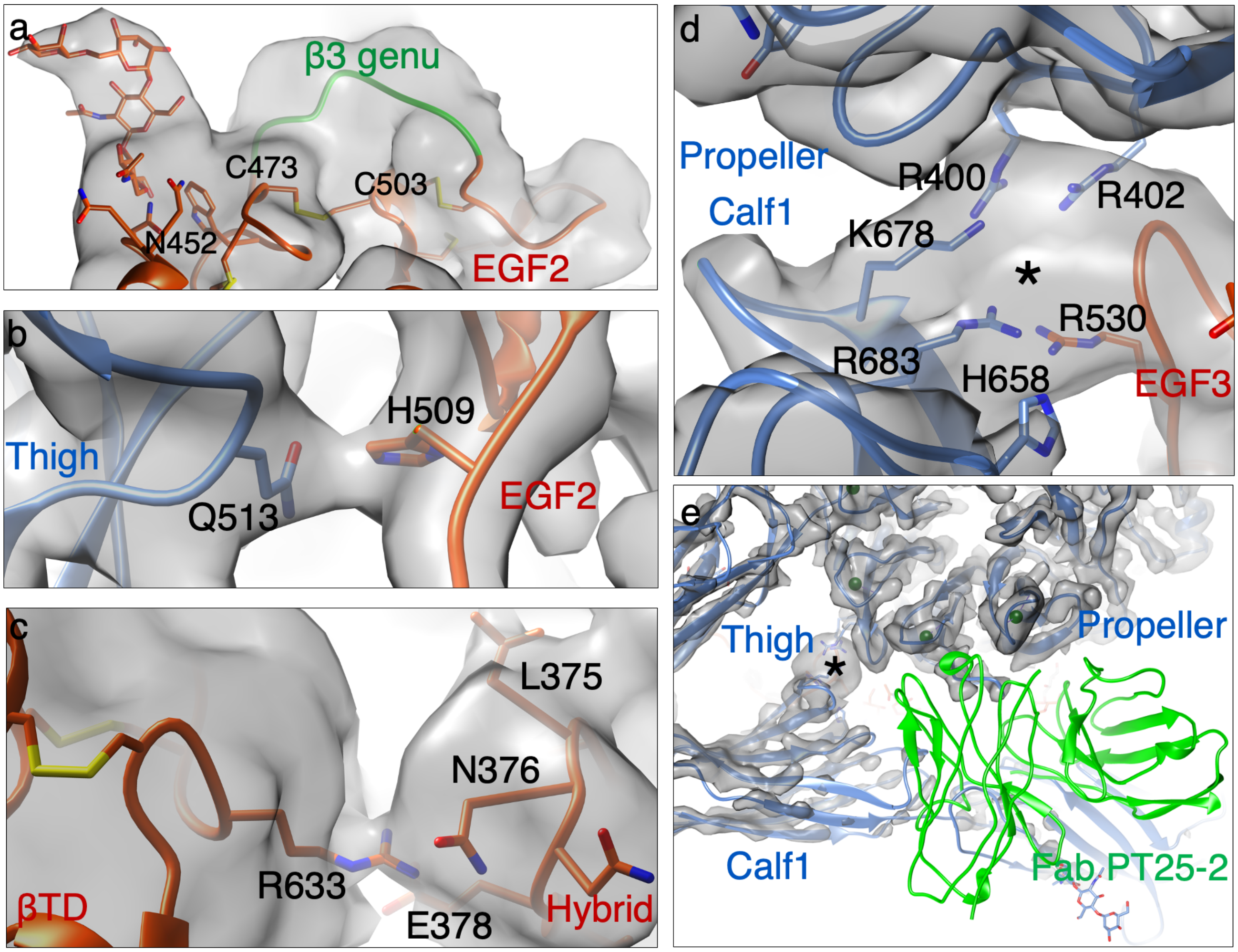

Supplementary Fig. 4. Structural elements observed in the full-length bent state. a) Cryo-EM density defining the structure of the β3 genu (shown as a green ribbon) of EGF2 (E476-Q483). The cysteine pair (C473 and C503) and N452 are labeled. The rest of the β3 subunit in this panel and in panels b-d are shown in orange. b) EM density demonstrating interactions between H509 of the β3 EGF2 domain and Q513 of the αIIb thigh domain (light blue ribbon shown here and in panels d and e). c) Interactions of a mixed nature between the βTD and the hybrid domain. Two regional disulfides in the βTD are shown. Densities for the other contacts between EGF2-PSI (S543-R8), EGF3-calf1 (Y556-R661), and βTD-calf2 (T603-R751) are not included in this figure. d) The new density (asterisk) making substantial contacts with R400 and R402 of the αIIb propellor domain, K678, R683, and H658 of the αIIb calf1 domain, and R530 of the β3 EGF3 domain. The density is strong enough to indicate that it is well-ordered. This feature, in addition to the basic coordinating residues, suggests that it may be composed of sulfate- or phosphate-containing molecules, such as a sulfonated glycosaminoglycan. e) The proximity of the new density (asterisk) to the binding site for the activating Fab PT25-2 (in green) is shown. Interactions maintaining this feature might well be disrupted by the antibody.

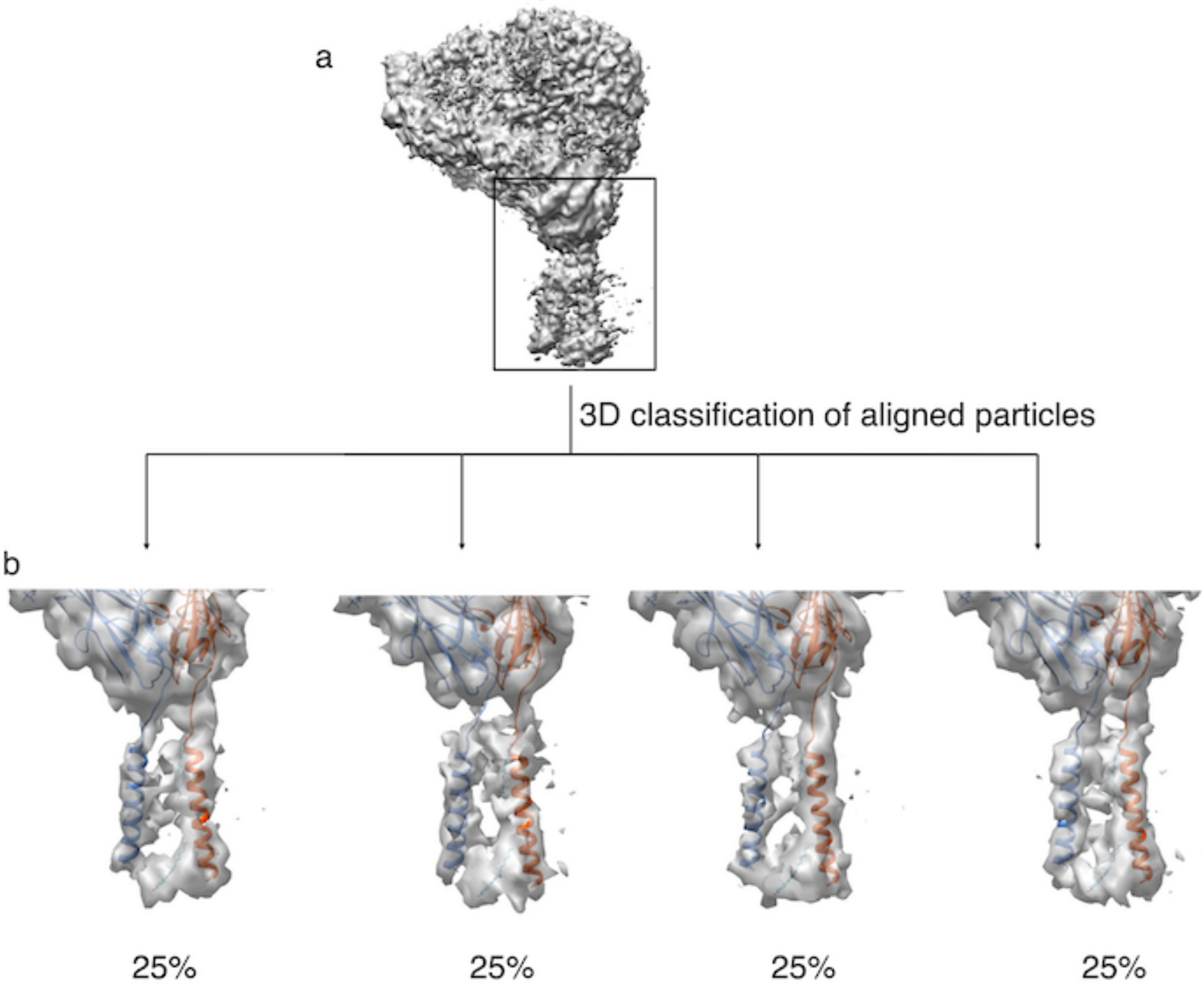

Supplementary Fig. 5. Outline of 3D classification analysis of the TM domains. a) The entire map with a box indicating the approximate area masked out for focused classification. b) Results of 3D classification of the masked region. αIIb is in light blue, and β3 is in orange. The densities of the cholesterol molecules (presented as shown in Fig. 2) are found in each of the four maps.

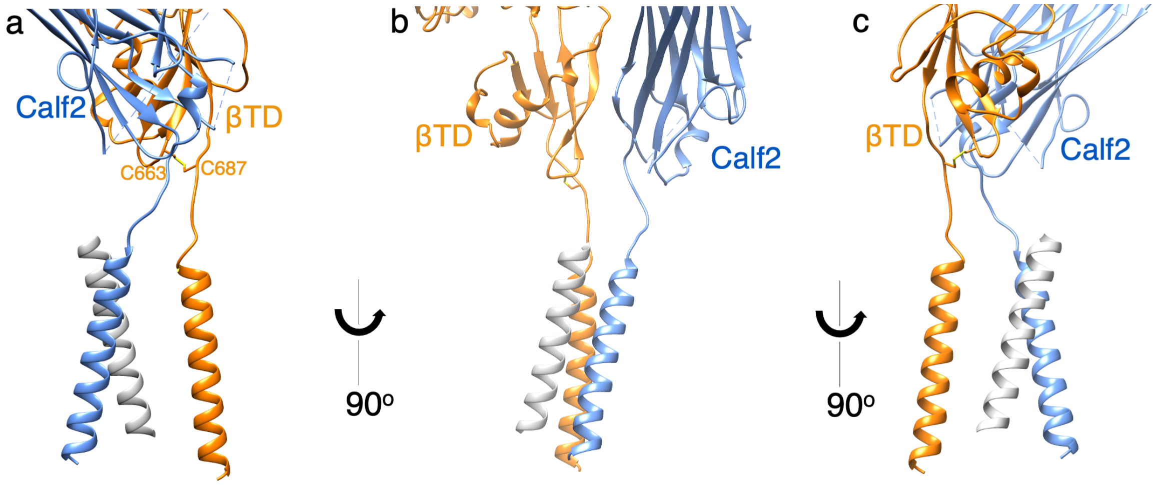

Supplementary Fig. 6. Separation of the TM domains of full-length inactive bent αIIbβ3. αIIb and β3 are in light blue and orange, respectively. The αIIb TM helix of the NMR structure of the associated synthetic αIIb and β3 TM peptides (2k9J) determined in bicelles, has been aligned with the corresponding αIIb TM helix of the EM structure. The Cαs of residues 693-717 of the β3 TM helix of the EM structure are displaced relative to the respective residues in the NMR structure (light gray) with an RMSD of 16.54 Å. The lower segments of calf2 and βTD are shown, as is the disulfide bond (in yellow) at the bottom of βTD. The orientation in (a) is the same as in Fig. 1a. (b) is rotated 90º relative to (a), and (c) is rotated 90º relative to (b).

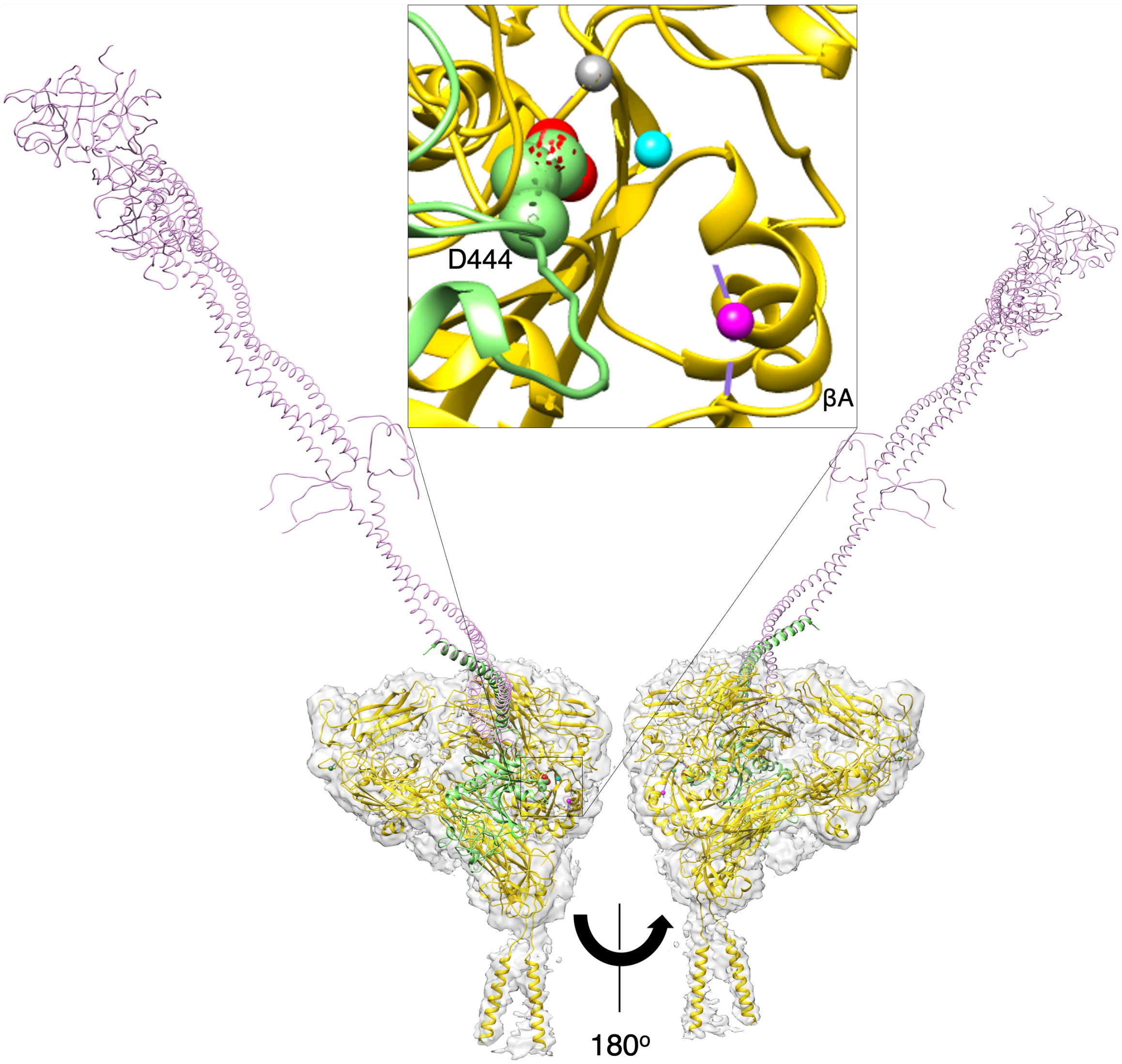

Supplementary Fig. 7. The αIIbβ3 ligand binding site in the cryo-EM bent structure is fully accessible to large ligands. The αIIbβ3 structure (yellow ribbon) in the EM density has been fitted with a model of a fibrinogen dimer. The γ C-terminus of the fibrinogen crystal structure (pdb id 3EIO, shown in magenta) has been extended with an AlphaFold model (AlphaFold DB C9JEU5, shown in green) bound to the integrin ligand binding site. *Inset*, a closeup of the ligand binding site showing coordination of the Mg^{2+} ion at MIDAS (cyan sphere) with the fibrinogen ligand D444 (shown in spheres with carbons in green and oxygens in red). Metal ions at ADMIDAS and LIMBS are in magenta and gray spheres, respectively.

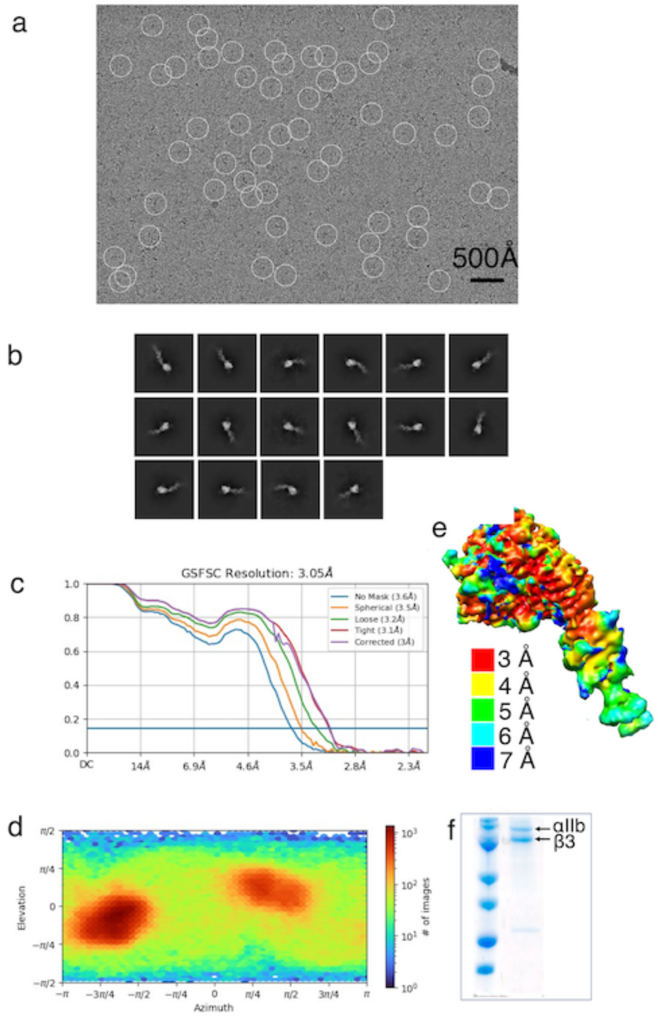

Supplementary Fig. 8. EM processing of αIIbβ3 bound to eptifibatide. a) A representative cryo-EM micrograph. Selected particles used in the final structure are indicated with white circles. This micrograph is one of 9,742 micrographs with selected particles. The scale bar at the lower right shows 500Å. b) reference-free class averages generated from selected particles. Domains attributable to regions beyond the integrin head cannot be identified in the averages. Due to preferential orientation, integrin class averages resemble one another (see panel (d) below). c) Fourier-shell correlation curves for the two half maps for the final round of refinement. The 0.143 cutoff criterion has been used to determine the resolution. d) Heat map showing the orientation distribution of particles. Euler angles are indicated along the X and Y coordinates. Color encoding for particle numbers is shown on the right. The distinct red patches indicate a strong preferential orientation for two orientations separated by 180° in azimuth. e) a color-coded local resolution map of the integrin (in a similar orientation to that in Fig. 4a) is generated using the 0.143 cutoff criterion. Colors encode graded resolutions between 3Å (red) and 7 Å (blue). f) Coomassie stain of an uncropped 10% unreduced SDS-PAGE of eptifibatide-bound full-length αIIbβ3 in NCMNP7b. This is one of two gels run after data collection. Lane 1, molecular mass markers (in kDa) are 250, 150, 100, 75, 50, 37.5, 25, and 20. Lane 2, eptifibatide-bound full-length αIIbβ3. The intact αIIb and β3 subunits are indicated by arrows. A faint band of undetermined identity is seen at ~28kDa.

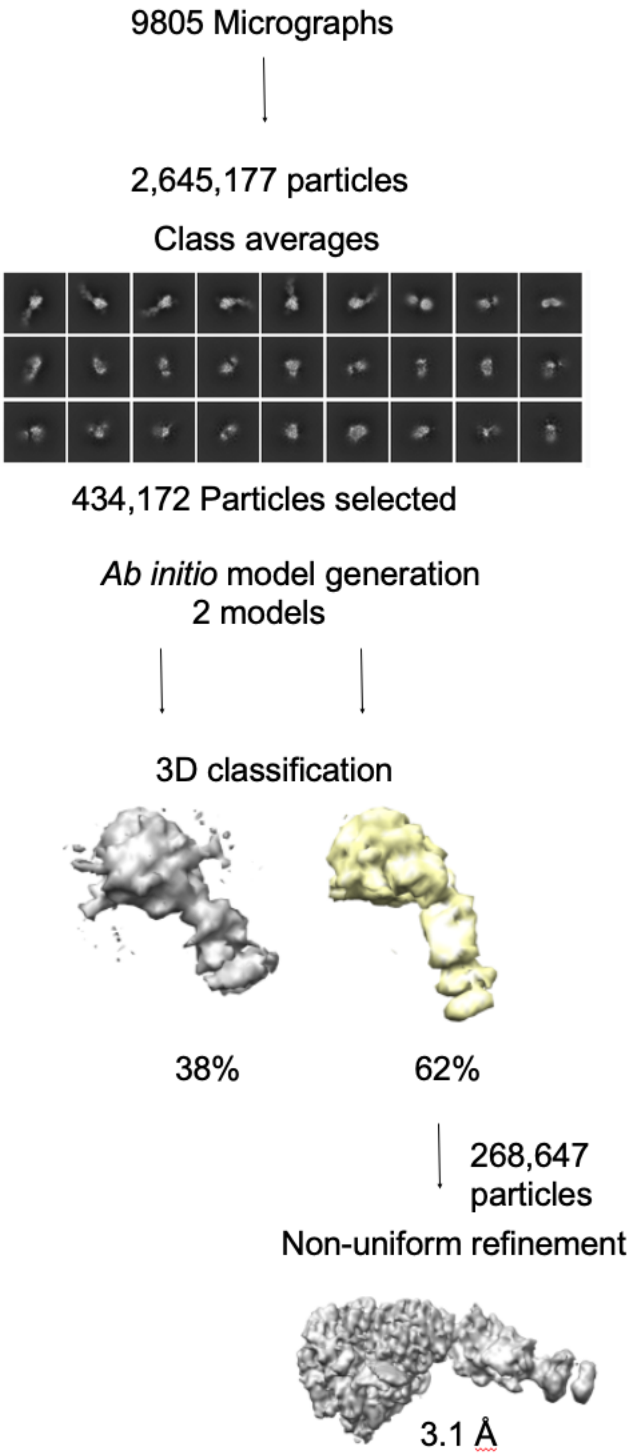

Supplementary Fig. 9. Flowchart for EM analysis of eptifibatide-bound full-αIIbβ3 with Cryosparc. Flowchart for processing of the integrin/drug complex. 27 out of 50 class averages are shown, six of which were used for *ab initio* model and further processing. The remaining details are provided in the methods section. The final map at 3.1 Å is displayed with the static mask used for refinement.

Supplementary Table 1. Cryo-EM data collection, refinement, and validation statistics

	(EMDB- 40989)<br>(PDB 8T2V)	(EMDB-40988)<br>(PDB 8T2U)
**Data collection and processing**		
Magnification	81,000	81.000
Voltage (kV)	300	300
Electron exposure (e–/Å^2)	50	50
Defocus range (μm)	-2.5 to -1.0	-2.2 to -1.2
Pixel size (Å)	1.08	1.08
Symmetry imposed	C1	C1
Initial particle images (no.)	4,477,836	2,645,177
Final particle images (no.)	117,126	268,647
Map resolution (Å)	3.4	3.1
FSC threshold	0.143	0.143
Map resolution range (Å)	2.4 - 7	2.7 - 7
**Refinement**		
Initial model used (PDB code)	3FCS and 2K9J	2VDN
Model resolution (Å)		
FSC threshold	0.143	0.143
Model resolution range (Å)	3.38	3.1
Map sharpening *B* factor (Å^2)	0	0
Model composition		
Non-hydrogen atoms	13134	6553
Protein residues	1664	822
Ligands	0	1
B factors (Å^2)		
Protein	199.55	173
Ligand		346.8
R.m.s. deviations		
Bond lengths (Å)	0.015	0.015
Bond angles (°)	1.976	1.804
Validation		
MolProbity score	2.68	2.98
Clashscore	11	29.82
Poor rotamers (%)	5.5	4.48
Ramachandran plot		
Favored (%)	90	90.8
Allowed (%)	9	8.61
Disallowed (%)	1	0.62

Supplementary Table 2. N-glycosylation sites and types in the bent structure

Integrin	Residue	Domain	Visible tree
αIIb	N15	Propeller	NAG*
αIIb	N249	Propeller	NAG
αIIb	N570	Thigh	NAG
αIIb	N680	Calf1	NAG
αIIb	N931	Calf2	NAG-NAG-MAN#
β3	N99	Hybrid	NAG-NAG
β3	N320	βA	NAG-NAG-MAN-MAN
β3	N452	EGF1	NAG-NAG-MAN
β3	N371	Hybrid	NAG-NAG- MAN-MAN-MAN
β3	N559	EGF3	NAG-NAG-MAN
β3	N654	βTD	NAG

* NAG, N-acetyl-D-glucosamine. # MAN, D-mannose
